# Technologies used in Parkinson’s disease: a meta-analysis of their effect on health-related quality of life

**DOI:** 10.1007/s00415-026-13749-6

**Published:** 2026-03-12

**Authors:** Cuma Fidan

**Affiliations:** Faculty of Health Sciences, Department of Healthcare Management, Muş Alparslan University, 49250 Muş, Turkey

**Keywords:** Health technologies, Health-related quality of life, Meta-analysis, Parkinson’s disease

## Abstract

**Objective:**

The results of randomised controlled trials (RCTs) and meta-analyses in the literature on whether technologies used in Parkinson’s disease (PD) improve health-related quality of life (HRQoL) are varied, making it difficult to reach a definitive conclusion. Therefore, the aim was to investigate the effect of technologies used in PD on HRQoL according to moderator variables.

**Methods:**

The mean effect size was calculated using either the fixed-effects or random-effects model. Assessment timepoints, technology and scale types were used as moderator variables. Technology types: Mobile health (mHealth), robotic-assisted, telerehabilitation, virtual reality and wearable technologies. The Egger’s regression method was used to assess publication bias. The quality assessment used the risk of bias (RoB) 2 method. The results of the meta-analysis were evaluated clinically and statistically.

**Results:**

The meta-analysis included 34 RCTs. RCTs were published in the form of thesis and article publications between 2012 and 2025. The control group had 709 patients, the experimental group 759. The RoB 2 method indicates that the majority of RCTs are low risk of bias. According to the Egger’s regression method, there is no publication bias. According to the results of the meta-analysis, mHealth, robotic-assisted, telerehabilitation, virtual reality and wearable technologies used in PD have the potential to improve patients’ HRQoL.

**Conclusion:**

The technologies used in PD have the potential to improve patients’ HRQoL. These results could provide significant opportunities for effectively managing the disease and its treatment, as well as improving patients’ daily living activities.

## Introduction

In Parkinson’s disease (PD), mobile health (mHealth), robotic-assisted, telerehabilitation, virtual reality and wearable technologies can be used to improve patients’ health-related quality of life (HRQoL) [1–5]. mHealth technologies aim to improve patients’ HRQoL with PD by encouraging regular exercise and improving treatment adherence and management of motor and non-motor symptoms [5–8]. Robotic-assisted technologies aim to improve patients’ HRQoL with PD by encouraging regular exercise and developing cognitive, motor and functional skills. They also aim to reduce gait disorders and postural instability [2, 9–12]. Telerehabilitation technologies aim to reduce the severity of motor symptoms in patients with PD, thereby improving upper extremity motor function and reducing postural instability. These technologies can also improve clinical outcomes by encouraging regular exercise and improving cognitive function and balance. Furthermore, they can increase walking speed and prevent falls, thereby improving activities of daily living and enhancing HRQoL [1, 13–21]. Virtual reality technologies aim to improve patients’ HRQoL with PD by reducing gait disturbances and improving balance to prevent falls. They also aim to develop cognitive and upper extremity motor functions and increase limb muscle strength [3, 22–32]. Wearable technologies aim to improve patients’ HRQoL with PD by reducing gait disturbances and postural instability, monitoring motor fluctuations continuously, adjusting medication doses correctly, and managing symptoms more effectively [4, 33, 34].

In summary, these technologies can be used in PD to improve patients’ HRQoL by encouraging regular exercise; developing cognitive, motor and functional skills; reducing symptoms, improving balance and preventing falls. Assessments of HRQoL in these settings may offer significant opportunities for effectively managing the disease and treatment, as well as improving patients’ daily living activities. Conversely, the results of randomised controlled trials (RCTs) [1–5, 8, 12, 18, 25, 33] and meta-analyses [35, 36] in the literature on whether technologies used in PD improve HRQoL are varied, making it difficult to reach a definitive conclusion. These RCTs use the following technologies: mHealth, robotic-assisted, telerehabilitation, virtual reality, and wearable technologies. Meta-analysis studies, however, are limited to RCTs on the use of virtual reality technology [35, 36]. These circumstances highlight the need for a comprehensive meta-analysis to generate robust and generalisable results. For these reasons, the aim of this study was to investigate the effect of technologies used in PD on HRQoL, according to moderator variables, using meta-analysis method. The following fundamental research question has been identified based on the aim of the study.

Primary research question: What effect do technologies used in PD have on HRQoL, according to moderator variables?

## Methods

### Reporting

The PRISMA (Preferred Reporting Items for Systematic Reviews and Meta-Analysis) guideline was used to report the meta-analysis study [37].

### Search strategy

A search was conducted in Web of Science, PubMed, Cochrane Library, ProQuest, Council of Higher Education (CoHE) Thesis Centre, TR Index, and DergiPark databases, up to January 11, 2026. The following Boolean search formula in Turkish and English was used for the searches:

- intitle: (“telerehabilitation” OR “virtual reality” OR “augmented reality” OR “wearable technology” OR “sensor” OR “mHealth” OR “mobile health” OR “robotic” OR “telemedicine” OR “telehealth” OR “technology” OR “deep brain stimulation” OR “artificial intelligence” OR “deep learning” OR “machine learning”) AND (“Parkinson’s disease” OR “quality of life” OR “randomised controlled trial”)
- intitle: (“telerehabilitasyon” VEYA “sanal gerçeklik” VEYA “artırılmış gerçeklik” VEYA “giyilebilir teknoloji” VEYA “sensör” VEYA “mobil sağlık” VEYA “robotik” VEYA “teletıp” VEYA “telesağlık” VEYA “teknoloji” VEYA “derin beyin stimülasyonu” VEYA “yapay zekâ” VEYA “derin öğrenme” VEYA “makine öğrenme”) VE (“Parkinson hastalığı” VEYA “yaşam kalitesi” VEYA “randomize kontrol çalışması”)

### Study selection

The inclusion criteria are as follows: (1) patients who have received a PD diagnosis; (2) patients in the experimental group (EG) who have undergone any technological intervention, and patients in the control group (CG) who have not undergone any technological intervention; (3) patients’ HRQoL having been assessed in both the EG and CG after the technological intervention (post-intervention), or patients’ HRQoL having been assessed in the EG during the pre-intervention, post-intervention and/or follow-up periods; (4) RCTs; (5) studies in Turkish and/or English; (6) full-text studies; and (7) studies reporting the statistics necessary to calculate the effect size.

The exclusion criteria are as follows: (1) patients who have not received a PD diagnosis; (2) patients in the EG who have not received any technological intervention and patients in the CG who have received any technological intervention; (3) patients’ HRQoL having not been assessed after technological intervention in the EG and CG (post-intervention), or patients’ HRQoL having not been assessed in the EG during the pre-intervention, post-intervention and/or follow-up periods; (4) studies other than RCTs; (5) studies in different languages; (6) abstract studies or studies whose full text could not be accessed; (7) studies that did not report the statistics necessary to calculate the effect size; and (8) duplicate studies.

The studies reviewed in the meta-analysis were assessed according to these selection criteria across four time periods. This corrected differences in the selection of studies, thus preventing researcher bias.

### Data extraction

The following data were obtained from the included studies: authors, study title, database, year of publication, language of publication, research design, technology type, scale type, assessment timepoints, post-intervention and follow-up assessment duration (in weeks), statistical data, and quality assessment results. To correct coding discrepancies, enhance data accuracy and prevent researcher bias, the author verified these data across four different time periods.

### HRQoL scales

The Short Form (SF)-36, SF-12, Parkinson’s Disease Questionnaire (PDQ)-39 and PDQ-8 scales were used to assess patients’ HRQoL in the studies included in the meta-analysis. The SF-36 scale comprises eight subscales. The SF-12 scale is a shorter version of the SF-36 scale. Scores on the SF-36 and SF-12 scales range from 0 to 100. A high score indicates a good health status [30, 31]. The PDQ-39 scale comprises eight subscales. The PDQ-8 is a shorter version of the PDQ-39 scale. Scores on the PDQ-39 and PDQ-8 scales range from 0 to 100. A low score indicates a good health status [1, 3].

### Meta-analysis

The random effect model was used to calculate the mean effect size (M) if the between-studies variance (Ʈ^2^) and the amount of heterogeneity (I^2^) were not equal to zero. The fixed effect model was used if Ʈ^2^ and I^2^ were equal to zero. To prevent differences in evaluation according to the SF and PDQ scales, the SF scale means were multiplied by −1 [38]. The moderator variables were determined as the assessment timepoints (pre-intervention, post-intervention, and follow-up periods), and the technology and scale types (mHealth, robotic-assisted, telerehabilitation, virtual reality, and wearable technologies; SF-36, SF-12, PDQ-39, and PDQ-8 scales). The meta-analyses used these variables for the EG vs. CG, and EG comparisons. The Comprehensive Meta-Analysis (CMA v. 4) programme was used to analyse the data.

### Publication bias

The publication bias analysis was performed using the Egger’s regression method. The method states that if a p-value is greater than 0.05, it indicates the absence of publication bias (p > 0.05) [39].

### Evaluation of clinical significance

The distribution-based method was used to calculate threshold values (Minimal Clinically Important Difference − MCID) in order to evaluate clinical significance [40]. Accordingly, a separate MCID has been calculated for each meta-analysis. If the M values are greater than the MCIDs, this indicates clinical significance (|M|> MCID) [38].

### Quality assessment

The risk of bias (RoB) 2 method was used to assess the quality of RCTs. The RoB 2 method consists of six domains: (1) the randomisation process, (2) deviations from the intended intervention, (3) missing outcome data, (4) measurement of the outcome, (5) selection of the reported result, and (6) overall risk of bias. Each domain is assessed as having a “low risk of bias”, “some concerns”, or “high risk of bias” [41]. To correct for assessment differences, enhance the accuracy of the data, and thereby prevent researcher bias, the quality assessments of the included RCTs were conducted across four different time periods.

## Results

### Study selection

**Fig. 1:**
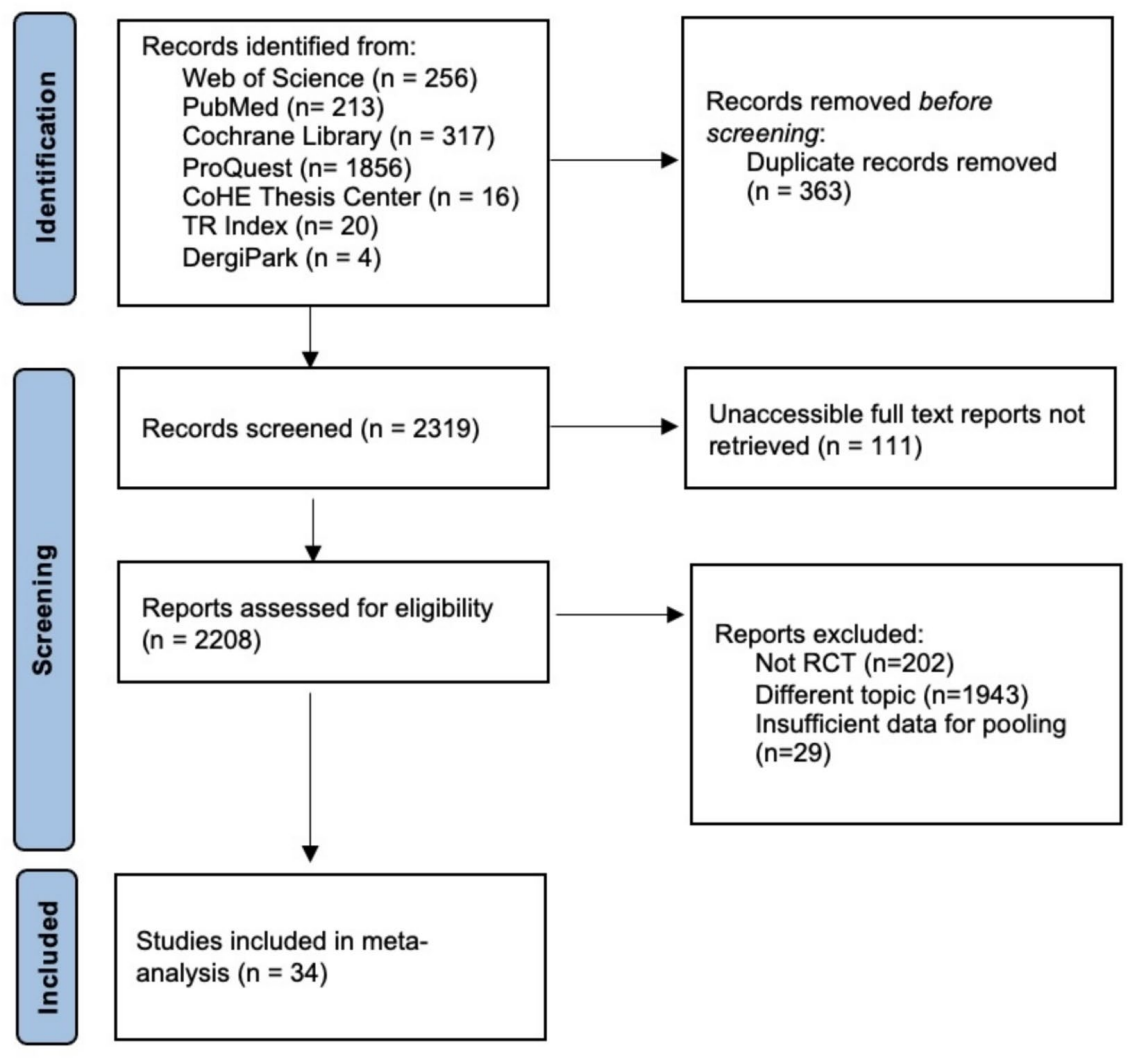
Flow diagram

A total of 2682 studies were identified through the screening process. Of these, 363 duplicate studies were excluded. Subsequently, 111 studies whose full text could not be accessed were excluded. Following a full-text review and assessment, 2174 of the remaining 2208 studies were excluded. In the end, 34 RCTs that met the inclusion criteria were included in the meta-analysis (Fig. 1).

### Study characteristics

The study sample consists of 34 RCTs that met the selection criteria from among 2682 publications (Fig. 1). The studies were published between 2012 and 2025 in the form of thesis and article publications in Turkish and/or English. They were conducted using an RCT study design. A total of 759 patients with PD were included in the EG, and 709 patients with PD were included in the CG. The technology types used in RCTs include mHealth, robotic-assisted, telerehabilitation, virtual reality and wearable technologies. The scale types that are used are the SF-36, SF-12, PDQ-39 and PDQ-8 scales (Table 1).

**Table 1:** Characteristics of the included studies

No	Author(s)	Year	Publication type	Research design	Technology type	Scale	Post-intervention (week)	Follow-up (week)	EG (N)	CG (N)	Follow-up (EG-N)	Post-intervention (EG-N)	Pre-intervention (EG-N)
1	Ayaz 2021	2021	Thesis	RCT	Virtual reality	PDQ-39	4		10	10		10	10
2	Benli 2024	2024	Thesis	RCT	Virtual reality	PDQ-8	8		22	22		22	22
3	Capecci et al., 2019	2019	Article	RCT	Robotic-assisted	PDQ-39	4		48	48		48	48
4	Carda et al., 2012	2012	Article	RCT	Robotic-assisted	SF-12	4	24	15	15	15	15	15
5	Carpinella et al., 2017	2017	Article	RCT	Wearable technology	PDQ-39	7	4	17	20	17	17	17
6	Çakır, 2023	2023	Thesis	RCT	Telerehabilitation	PDQ-39	8					30	30
7	Çetin, 2024	2024	Thesis	RCT	Virtual reality	PDQ-39	8		10	10		10	10
8	Da Silva et al., 2023	2023	Article	RCT	Virtual reality	PDQ-39	7	11	18	20	18	18	18
9	Eldemir, 2023	2023	Thesis	RCT	Telerehabilitation	PDQ-8	6		15	15		15	15
10	Eldemir et al., 2023	2023	Article	RCT	Telerehabilitation	PDQ-8	6		15	15		15	15
11	Ellis et al., 2019	2019	Article	RCT	mHealth	PDQ-39	48		26	25		26	26
12	Ferraz et al., 2018	2018	Article	RCT	Virtual reality	PDQ-39	32		20	22		20	20
13	Fiorenzato et al., 2025	2025	Article	RCT	Virtual reality	PDQ-39	4	8	15	13	15	15	15
14	Gandolfi et al., 2017	2017	Article	RCT	Telerehabilitation	PDQ-8	7	11	38	38	38	38	38
15	Ge et al., 2024	2024	Article	RCT	Telerehabilitation	PDQ-39	4		90	100		90	90
16	Ginis et al., 2016	2016	Article	RCT	mHealth	SF-36	6	10	20	18	20	20	20
17	Gryfe et al., 2022	2022	Article	RCT	Robotic-assisted	PDQ-39	8		13	13		13	13
18	Hajebrahimi, 2020	2020	Thesis	RCT	Virtual reality	PDQ-39	4		11	13		11	11
19	Johnson et al., 2024	2024	Article	RCT	Telerehabilitation	PDQ-39	4		5	8		5	5
20	Kaya Aytutuldu, 2021	2021	Thesis	RCT	Telerehabilitation	PDQ-39	4					16	16
21	Kaya Aytutuldu et al., 2024	2024	Article	RCT	Telerehabilitation	PDQ-39	4					17	17
22	Liao et al., 2015	2015	Article	RCT	Virtual reality	PDQ-39	6	10	12	12	12	12	12
23	Maggio et al., 2025	2025	Article	RCT	Telerehabilitation	PDQ-8	8		10	10		10	10
24	Maranesi et al., 2022	2022	Article	RCT	Virtual reality	SF-12	5		16	14		16	16
25	Park et al., 2022	2022	Article	RCT	mHealth	PDQ-39	16		20	23		20	20
26	Pazzaglia et al., 2020	2020	Article	RCT	Virtual reality	SF-36	6		25	26		25	25
27	Raciti et al., 2025	2025	Article	RCT	Robotic-assisted	PDQ-39	8		20	20		20	20
28	Rodríguez-Fuentes et al., 2024	2024	Article	RCT	Virtual reality	PDQ-39	12		30	22		30	30
29	Rodríguez-Molinero et al., 2025	2025	Article	RCT	Wearable technology	PDQ-39	26		50	50		50	50
30	Spina et al., 2021	2021	Article	RCT	Robotic-assisted	PDQ-39	4	8	11	11	11	11	11
31	Theodoros et al., 2016	2016	Article	RCT	Telerehabilitation	PDQ-39	4		15	16		15	15
32	Volpe et al., 2014	2014	Article	RCT	Wearable technology	PDQ-39	9	17	20	20	20	20	20
33	Yang et al., 2016	2016	Article	RCT	Virtual reality	PDQ-39	6	8	11	12	11	11	11
34	Yılmaz, 2024	2024	Thesis	RCT	mHealth	PDQ-39	4	12	48	48	48	48	48

RCT: randomised controlled trial, mHealth: mobile health, PDQ: Parkinson’s disease questionnaire, SF: short form, EG: experimental group, CG: control group, N: sample size

### Results of the quality assessment

The RoB 2 method’s quality assessment indicates low risk of bias in 23 studies, and some concerns in 11 studies (Table 2).

**Table 2:**
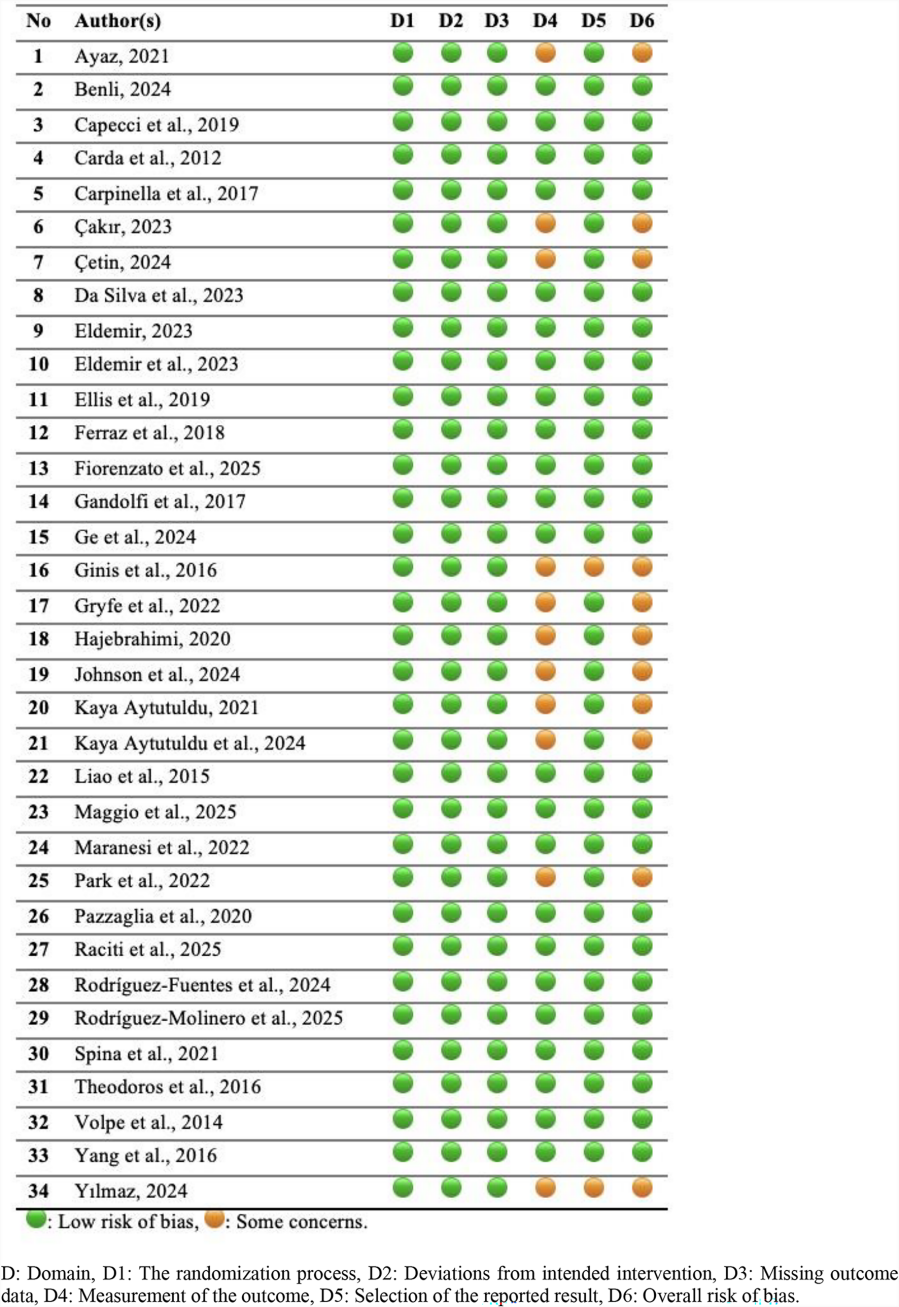
Results of the quality assessment

### Results of the publication bias analysis

According to the Egger’s regression method, there is no publication bias in the meta-analyses of EG & CG (post-intervention) (t = 1.18; p = 0.12), post- & pre-interventions (EG) (t = 1.06; p = 0.15), follow-up & pre-intervention (EG) (t = 1.79; p = 0.06), and follow-up & post-intervention (EG) (t = 1.65; p = 0.07).

### Results of the meta-analysis

#### Results of the meta-analysis on the effect of mHealth technology on HRQoL

In mHealth technology, EG patients have statistically and clinically better HRQoL than CG patients (M = −0.56, 95% CI [−1.08, −0.05], p_M_ < 0.05, |M|> MCID). According to the PDQ-39 scale, they also have clinically better HRQoL (95% CI, p_M_ > 0.05, |M|> MCID). Other analysis results suggest that mHealth technology clinically improves patients’ HRQoL (95% CI, p_M_ > 0.05, |M|> MCID) (Tables 3 and 4).

**Table 3:** Results of the meta-analysis according to assessment timepoints and technology types

Assessment timepoints	Technology type	K	M	95% CI		z	p_M_	Q	*df*	p_Q_	I^2^	Ʈ^2^	MCID
EG & CG (Post-intervention)	mHealth	4	−0.56	−1.08	−0.05	−2.13	0.033^a^	10.92	3	0.012	72.54	0.20	0.13^b^
EG & CG (Post-intervention)	Robotic-assisted	5	0.02	−0.34	0.38	0.12	0.905	6.49	4	0.165	38.37	0.06	0.09
EG & CG (Post-intervention)	Telerehabilitation	7	−0.06	−0.28	0.15	−0.58	0.559	6.56	6	0.364	8.48	0.01	0.055^b^
EG & CG (Post-intervention)	Virtual reality	12	−0.36	−0.68	−0.04	−2.24	0.025^a^	27.00	11	0.005	59.26	0.18	0.08^b^
EG & CG (Post-intervention)	Wearable technology	3	−3.05	−8.62	2.53	−1.07	0.284	0.88	2	0.645	0.00	0.00	1.42^b^
Post- & pre- interventions (EG)	mHealth	4	−0.27	−0.70	0.15	−1.27	0.204	7.34	3	0.062	59.14	0.11	0.11^b^
Post- & pre- interventions (EG)	Robotic-assisted	5	−0.24	−0.50	0.03	−1.77	0.076	1.82	4	0.768	0.00	0.00	0.07^b^
Post- & pre- interventions (EG)	Telerehabilitation	10	−0.64	−0.99	−0.29	−3.58	0.000^a^	28.26	9	0.001	68.15	0.20	0.09^b^
Post- & pre- interventions (EG)	Virtual reality	12	−0.25	−0.63	0.14	−1.23	0.217	41.12	11	0.000	73.25	0.34	0.10^b^
Post- & pre- interventions (EG)	Wearable technology	3	−6.70	−18.58	5.18	−1.11	0.269	6.95	2	0.031	71.21	76.84	3.03^b^
Follow-up & pre-intervention (EG)	mHealth	2	−0.61	−1.66	0.44	−1.14	0.256	7.76	1	0.005	87.12	0.50	0.27^b^
Follow-up & pre-intervention (EG)	Robotic-assisted	2	−0.41	−0.94	0.13	−1.50	0.134	1.00	1	0.318	0.00	0.00	0.14^b^
Follow-up & pre-intervention (EG)	Virtual reality	4	−6.58	−13.03	−0.12	−2.00	0.046^a^	2.32	3	0.509	0.00	0.00	1.65^b^
Follow-up & pre-intervention (EG)	Wearable technology	2	−5.16	−15.48	5.15	−0.98	0.326	1.08	1	0.298	7.66	4.66	2.63^b^
Follow-up & post-intervention (EG)	mHealth	2	−0.25	−0.61	0.10	−1.40	0.163	1.07	1	0.301	6.37	0.00	0.09^b^
Follow-up & post-intervention (EG)	Robotic-assisted	2	−0.07	−0.60	0.45	−0.28	0.781	0.00	1	0.956	0.00	0.00	0.13
Follow-up & post-intervention (EG)	Virtual reality	4	−1.79	−7.78	4.20	−0.59	0.558	0.34	3	0.953	0.00	0.00	1.53^b^
Follow-up & post-intervention (EG)	Wearable technology	2	7.61	−2.75	17.96	1.44	0.150	0.28	1	0.596	0.00	0.00	2.64^b^

EG: experimental group, CG: control group, mHealth: mobile health, k: number of studies, M: mean effect size, CI: confidence interval, z: normal standard deviation, p_M_: M’s significance value, Q: Cochran’s test for heterogeneity, p_Q_: Q’s significance value, *df*: degree of freedom, I^2^: amount of heterogeneity, Ʈ^2^: between-study variance, MCID: minimal clinically important difference

^a^p_M_ < 0.05

^b^|M|> MCID

**Table 4:** Results of the meta-analysis according to assessment timepoints, scales and technology types

Assessment timepoints	Scale type	Technology type	k	M	95% CI		z	p_M_	Q	*df*	p_Q_	I^2^	Ʈ^2^	MCID
EG & CG (Post-intervention)	PDQ-39	mHealth	3	−7.15	−15.60	1.29	−1.66	0.097	14.99	2	0.001	86.66	45.95	2.15^b^
EG & CG (Post-intervention)	PDQ-39	Robotic-assisted	4	0.12	−9.98	10.23	0.02	0.981	7.21	3	0.066	58.36	59.81	2.58
EG & CG (Post-intervention)	PDQ-39	Telerehabilitation	3	0.50	−7.29	8.30	0.13	0.900	3.37	2	0.186	40.64	18.81	1.99
EG & CG (Post-intervention)	PDQ-8	Telerehabilitation	4	−1.02	−4.22	2.18	−0.62	0.533	3.62	3	0.306	17.12	2.36	0.82^b^
EG & CG (Post-intervention)	PDQ-39	Virtual reality	9	−7.42	−13.07	−1.77	−2.57	0.010^a^	29.83	8	0.000	73.18	43.92	1.44^b^
EG & CG (Post-intervention)	PDQ-39	Wearable technology	3	−3.05	−8.62	2.53	−1.07	0.284	0.88	2	0.645	0.00	0.00	1.42^b^
Post- & pre- interventions (EG)	PDQ-39	mHealth	3	−3.70	−10.32	2.92	−1.09	0.274	10.91	2	0.004	81.67	25.97	1.69^b^
Post- & pre- interventions (EG)	PDQ-39	Robotic-assisted	4	−5.43	−11.00	0.15	−1.91	0.057	1.40	3	0.706	0.00	0.00	1.42^b^
Post- & pre- interventions (EG)	PDQ-39	Telerehabilitation	6	−9.18	−15.28	−3.08	−2.95	0.003^a^	15.69	5	0.008	68.13	35.43	1.56^b^
Post- & pre- interventions (EG)	PDQ-8	Telerehabilitation	4	−5.29	−7.38	−3.20	−4.96	0.000^a^	0.49	3	0.922	0.00	0.00	0.53^b^
Post- & pre- interventions (EG)	PDQ-39	Virtual reality	9	−4.93	−8.50	−1.35	−2.70	0.007^a^	13.23	8	0.104	39.55	9.27	0.91^b^
Post- & pre- interventions (EG)	PDQ-39	Wearable technology	3	−6.70	−18.58	5.18	−1.11	0.269	6.95	2	0.031	71.21	76.84	3.03^b^
Follow-up & pre-intervention (EG)	PDQ-39	Virtual reality	4	−6.58	−13.03	−0.12	−2.00	0.046^a^	2.32	3	0.509	0.00	0.00	1.65^b^
Follow-up & pre-intervention (EG)	PDQ-39	Wearable technology	2	−5.16	−15.48	5.15	−0.98	0.326	1.08	1	0.298	7.66	4.66	2.63^b^
Follow-up & post-intervention (EG)	PDQ-39	Virtual reality	4	−1.79	−7.78	4.20	−0.59	0.558	0.34	3	0.953	0.00	0.00	1.53^b^
Follow-up & post-intervention (EG)	PDQ-39	Wearable technology	2	7.61	−2.75	17.96	1.44	0.150	0.28	1	0.596	0.00	0.00	2.64^b^

EG: experimental group, CG: control group, mHealth: mobile health, PDQ: Parkinson’s disease questionnaire, k: number of studies, M: mean effect size, CI: confidence interval, z: normal standard deviation, p_M_: M’s significance value, Q: Cochran’s test for heterogeneity, p_Q_: Q’s significance value, *df*: degree of freedom, I^2^: amount of heterogeneity, Ʈ^2^: between-study variance, MCID: minimal clinically important difference

^a^p_M_ < 0.05

^b^|M|> MCID

#### Results of the meta-analysis on the effect of robotic-assisted technology on HRQoL

In robotic-assisted technology, post-intervention and follow-up patients in the EG have clinically better HRQoL than pre-intervention patients (95% CI, p_M_ > 0.05, |M|> MCID). Other analysis results show that the use of robotic-assisted technology has no statistically or clinically significant improvement on HRQoL (95% CI, p_M_ > 0.05, |M|< MCID) (Tables 3 and 4).

#### Results of the meta-analysis on the effect of telerehabilitation technology on HRQoL

In telerehabilitation technology, post-intervention patients in the EG have statistically and clinically better HRQoL than pre-intervention patients (95% CI, p_M_ < 0.05, |M|> MCID). The PDQ-39 scale shows no statistical or clinical improvement in technology use HRQoL for EG patients compared to CG patients (95% CI, p_M_ > 0.05, |M|< MCID). However, the PDQ-8 scale shows clinical improvement in HRQoL (95% CI, p_M_ > 0.05, |M|> MCID) (Tables 3 and 4).

#### Results of the meta-analysis on the effect of virtual reality technology on HRQoL

In virtual reality technology, EG patients have statistically and clinically better HRQoL than CG patients, and follow-up patients in the EG have better HRQoL than pre-intervention patients (95% CI, p_M_ < 0.05, |M|> MCID). However, follow-up patients in the EG have clinically better HRQoL than post-intervention patients (95% CI, p_M_ > 0.05, |M|> MCID). Conversely, although post-intervention patients in the EG have clinically better HRQoL than pre-intervention patients (95% CI, p_M_ > 0.05, |M|> MCID), they also have statistically and clinically better HRQoL according to the PDQ-39 scale (M = −4.93, 95% CI [−8.50, −1.35], p_M_ < 0.05, |M|> MCID) (Tables 3 and 4).

#### Results of the meta-analysis on the effect of wearable technologies on HRQoL

In wearable technologies, EG patients have clinically better HRQoL than CG patients, and post-intervention and follow-up patients in the EG have better HRQoL than pre-intervention patients (95% CI, p_M_ > 0.05, |M|> MCID). On the other hand, post-intervention patients in the EG have clinically better HRQoL than follow-up patients (95% CI, p_M_ > 0.05, |M|> MCID) (Tables 3 and 4).

## Discussion and conclusion

The meta-analysis comprised 34 RCTs. These RCTs were published as theses and articles between 2012 and 2025. A total of 759 patients with PD were included in the EG, and 709 patients with PD were included in the CG. RCTs have used mHealth, robotic-assisted, telerehabilitation, virtual reality, and wearable technologies. HRQoL in patients with PD was assessed using the SF-36, SF-12, PDQ-39, and PDQ-8 scales. Conversely, the literature on meta-analysis studies is limited to the PDQ-39 scale and virtual reality technology [35, 36]. This situation shows that this is the most comprehensive meta-analysis study in the literature. The absence of publication bias enhances the validity and reliability of this meta-analysis study [39].

The following results were obtained in RCTs found in the literature: (1) The use of mHealth technology either improves [5] or does not improve HRQoL in patients with PD [6–8], (2) The use of robotic-assisted technology either improves [2, 9] or does not improve HRQoL in patients with PD [11, 12], (3) The use of telerehabilitation technology either improves [1, 13–15, 17, 19, 20] or does not improve HRQoL in patients with PD [18, 21], (4) The use of virtual reality technology either improves [3, 22–24, 26, 29, 31, 32] or does not improve HRQoL in patients with PD [25, 28, 30], and (5) The use of wearable technologies either improves [4] or does not improve HRQoL in patients with PD [33]. In meta-analyses in the literature, it has been concluded that virtual reality technology either improves [36] or has no improvement on HRQoL in patients with PD [35]. The results of the RCTs and the meta-analyses in the literature differ from one another. This meta-analysis concluded that mHealth, robotic-assisted, telerehabilitation, virtual reality, and wearable technologies have the potential to improve patients’ HRQoL. These results from the meta-analysis may present significant opportunities for the effective management of the disease and its treatment, as well as for improving patients’ daily living activities.

Taken together, the results of the meta-analyses conducted in the study suggest that mHealth, robotic-assisted, telerehabilitation, virtual reality, and wearable technologies generally improve patients’ HRQoL in a clinically and/or statistically significant manner. Conversely, it was only concluded in the EG & CG (post-intervention) and follow-up & post-intervention (EG) meta-analyses that robotic-assisted technology did not improve HRQoL in patients with PD, either clinically or statistically. However, post-intervention, only patients in the EG with wearable technology had a clinically better HRQoL than follow-up patients. These differences in the results of the meta-analyses are due to the different subscale assessments of the PDQ and SF scales, the small number of RCTs in which the SF scales were used, and the greater proportion of patients with lower HRQoL in the intergroup comparisons. This situation indicates that more RCTs are needed to increase statistical reliability and clinical significance.

This meta-analysis has some limitations. The first limitation relates to the RCTs that were included in the analysis. These are: (1) the limited number of RCTs assessing follow-up patients’ HRQoL, (2) the fact that technological interventions were carried out over very short periods in most RCTs, (3) the relatively small sample size in some RCTs, and (4) the limited number of RCTs involving mHealth, robotic-assisted, and wearable technologies. These limitations suggest that more RCTs are required. The second limitation relates to the analyses. In the study, the effect of technologies used in PD on HRQoL was analysed based on scale scores reported in the RCTs included in the analysis. The effect of technology use on HRQoL has been limited by assessment timepoints, technology, and scale type variables. Conversely, various technological factors could effect HRQoL. These variables include age, socioeconomic status, psychological health, disease stage, comorbidities, clinical findings, and access to and cost of technology. Another limitation regarding the analysis is that the post-intervention and follow-up assessments in the RCTs included in the analysis were conducted in different weeks. These differences in assessment times may both have an effect on patients’ HRQoL, and limit the comparability of meta-analysis results over time. For these reasons, conducting a meta-analysis to investigate the effect of these variables on HRQoL in technology use is recommended. The final limitation relates to the planning stage of the study. The study plans to investigate the effect of augmented reality, telemedicine, telehealth, deep brain stimulation, and artificial intelligence technologies on HRQoL. However, the limited number of studies conducted on these technologies, and the scarcity of RCTs in particular, has prevented analyses from being carried out. Therefore, it is recommended that RCTs be conducted to investigate the effect of augmented reality, telemedicine, telehealth, deep brain stimulation, and artificial intelligence technologies on HRQoL.

The meta-analysis included RCTs which reported some important limitations and technology-specific disadvantages [1, 3, 8, 9, 11, 12, 14, 17, 20, 22, 24, 25, 27]. Accordingly, factors such as short-term technological intervention, uncertainty regarding long-term effects and side effects, and small sample sizes come to the fore. These factors should be addressed by conducting RCTs.

In conclusion, the technologies used in PD have the potential to improve patients’ HRQoL. The results of this meta-analysis should be considered alongside various variables that may effect HRQoL when using technology, and the disadvantages specific to technology use.

## Limitations

The studies included in the meta-analysis were accessed via the following databases: Web of Science, PubMed, Cochrane Library, ProQuest, CoHE Thesis Centre, TR Index and DergiPark. The analysis included RCTs according to the selection criteria. Therefore, the study is limited to data obtained from the included RCTs. The effect of the technologies used in PD on HRQoL is limited by the variables of assessment timepoints, technology, and scale type. Therefore, it is recommended that meta-analyses are conducted on the different variables that may effect HRQoL when using technology (e.g. age, psychological health, disease stage, and clinical findings).

## Data Availability

The datasets relevant to this paper can be found in Table 1 and can be checked in the references. The data that support the findings of this study are available from the included studies.
